# Fitness Inference from Short-Read Data: Within-Host Evolution of a Reassortant H5N1 Influenza Virus

**DOI:** 10.1093/molbev/msv171

**Published:** 2015-08-04

**Authors:** Christopher J.R. Illingworth

**Affiliations:** ^1^Department of Genetics, University of Cambridge, Cambridge, United Kingdom

**Keywords:** influenza, fitness landscape, within-host evolution, linkage disequilibrium, selection

## Abstract

We present a method to infer the role of selection acting during the within-host evolution of the influenza virus from short-read genome sequence data. Linkage disequilibrium between loci is accounted for by treating short-read sequences as noisy multilocus emissions from an underlying model of haplotype evolution. A hierarchical model-selection procedure is used to infer the underlying fitness landscape of the virus insofar as that landscape is explored by the viral population. In a first application of our method, we analyze data from an evolutionary experiment describing the growth of a reassortant H5N1 virus in ferrets. Across two sets of replica experiments we infer multiple alleles to be under selection, including variants associated with receptor binding specificity, glycosylation, and with the increased transmissibility of the virus. We identify epistasis as an important component of the within-host fitness landscape, and show that adaptation can proceed through multiple genetic pathways.

## Introduction

The manner in which selection acts upon a population can be characterized in terms of a fitness landscape, which describes the fitness of each genotype in the population (Wright 1932). Fitness landscapes are of great interest due to the potential they convey to predict the future evolution of an evolutionary system (de Visser and Krug 2014). For example, characterization of the rates at which drug resistance mutations in HIV are acquired has enabled the computational optimization of combination therapies that will prolong the development of resistance (Beerenwinkel et al. 2005).

A prominent example of the use of inferred fitness values to predict evolution is that of the seasonal A/H3N2 influenza population. Phylogenetic models of the past development of the virus have been combined with sequence-based fitness models, or analyzed in their own right (Bush 1999; Luksza and Lässig 2014; Neher et al. 2014), to predict the strain most likely to be prevalent in the following influenza season. In this case, efficient strain prediction has the potential to improve the selection of candidates for vaccine development, conveying a significant benefit to human health.

Another scenario in which fitness effects have been highlighted as a key unknown factor is that of within-host influenza evolution (Russell et al. 2012). For example, the highly pathogenic H5N1 avian influenza virus is a zoonotic infection, occasionally infecting humans, but currently unable to transmit efficiently from person to person. The pandemic risk posed by the virus thus depends upon the potential for the virus to evolve greater transmissibility between human hosts (Morse et al. 2012). Such evolution would require the acquisition of genetic substitutions by the virus sufficient to change the viral phenotype, a process that could potentially occur within a single host.

Sequencing studies of within-host influenza evolution have been performed in multiple contexts, for example, comparing viral evolution in vaccinated and nonvaccinated hosts (Murcia et al. 2010, 2013), or examining the onset of drug resistance in a population grown in vitro (Foll et al. 2014). Considering H5N1 evolution, evolutionary experiments have shown that airborne-transmissible variants of H5N1 can be constructed directly under lab conditions (Imai et al. 2012), or can be evolved through repeated transmission of modified H5N1 virus (Herfst et al. 2012).

Despite the potential importance of the problem, current inference models for fitness effects in influenza are limited in their scope. In a case where a viral population is adapting to a novel environment, changes in the genetic structure of the population may be rapid, and complex. Due to the importance of host-virus interactions, approaches based upon in vitro data are likely not to provide a complete understanding (Hinkley et al. 2011). Large data sets describing the abundance of genotypes within the novel host are unlikely to be available (Ferguson et al. 2013). During adaptation, the viral population is likely to be far from evolutionary equilibrium, ruling out approaches based upon an equilibrium assumption (Seifert et al. 2014). A further challenge is posed by the importance of clonal interference in the evolution of influenza (Miralles et al. 1999; Strelkowa and Lässig 2012). In cases where only a single mutation is present in a gene, or where recombination is rapid, selection can be inferred from changes in single allele frequencies over time (Ganusov et al. 2011; Acevedo et al. 2014; Foll et al. 2014). Yet where linkage disequilibrium exists between selected alleles, such approaches are prone to error; an increase in frequency does not imply positive selection (Hill and Robertson 1966; Illingworth and Mustonen 2011). Methods for inferring selection in the presence of clonal interference have been described, but have previously been limited in their application, depending for example, upon complete haplotype data (Illingworth et al. 2014), or an underlying situation in which beneficial alleles arise in sequential fashion (Kessinger et al. 2013).

Here, we describe a novel method that uses short-read sequence data to begin to characterize the fitness landscape of the within-host evolution of influenza. Our method considers paired-end reads as partial haplotypes, which contain limited information about the extent of linkage disequilibrium between loci. Treating these haplotypes as noisy emissions from an inferred set of genome-length haplotypes, we construct a likelihood model by which we infer the extent to which selection has acted upon the virus.

We apply our method to data collected from two transmission experiments conducted using a reassortant H5N1 influenza virus in ferrets (Wilker et al. 2013), using our method to begin to characterize the fitness landscape of this virus. Previous studies of these experiments have examined the role of natural selection occurring both within-host, and in the transmission between hosts. Selection bottlenecks in the hemagglutinin (HA) gene have been observed in reassortant H5N1 viruses (Wilker et al. 2013) and during the experimental evolution of transmissibility, through a decrease in viral diversity (Wilker et al. 2013; Linster et al. 2014). Within-host, changes in allele frequency suggest that the evolution of the virus is not selectively neutral. However, the full role of selection in these experiments has not yet been characterized, such that scope remains for further deductions to be made from the available data. Based upon a conservative statistical approach, we identify selection acting upon multiple variants within the virus. Different pathways toward within-host adaptation are observed, resulting from differences in the standing variation present in different initial viral populations, and stochastic effects in the founding of an infection. We discuss the value of evolutionary experiments in predicting the future evolution of the H5N1 virus.

## Results

Data were analyzed from both simulated evolutionary systems, and from replica transmission experiments conducted between ferrets, reported in a previous publication (Imai et al. 2012). In the experiments, “index” ferrets were inoculated with a large dose of virus (10^6^ plaque forming units), then housed in adjacent isolator cages with uninfected, “contact” ferrets. Different reassortant viruses were used for each experiment, each including a modified H5 HA gene derived from the avian virus A/Vietnam/1203/2004, with other segments from the H1N1 virus A/California/04/2009. In the first experiment, the virus (denoted VN1203-HA(3)-CA04) contained three nonsynonymous substitutions in HA, associated with increased viral titer in ferret nasal turbinates (N158D) and human-type sialic acid receptor binding (N224K and Q226L). In the second experiment, the virus used (denoted VN1203-HA(4)-CA04) also contained the T318I substitution in HA, associated with improved transmission efficiency.

**Fig. 1.**
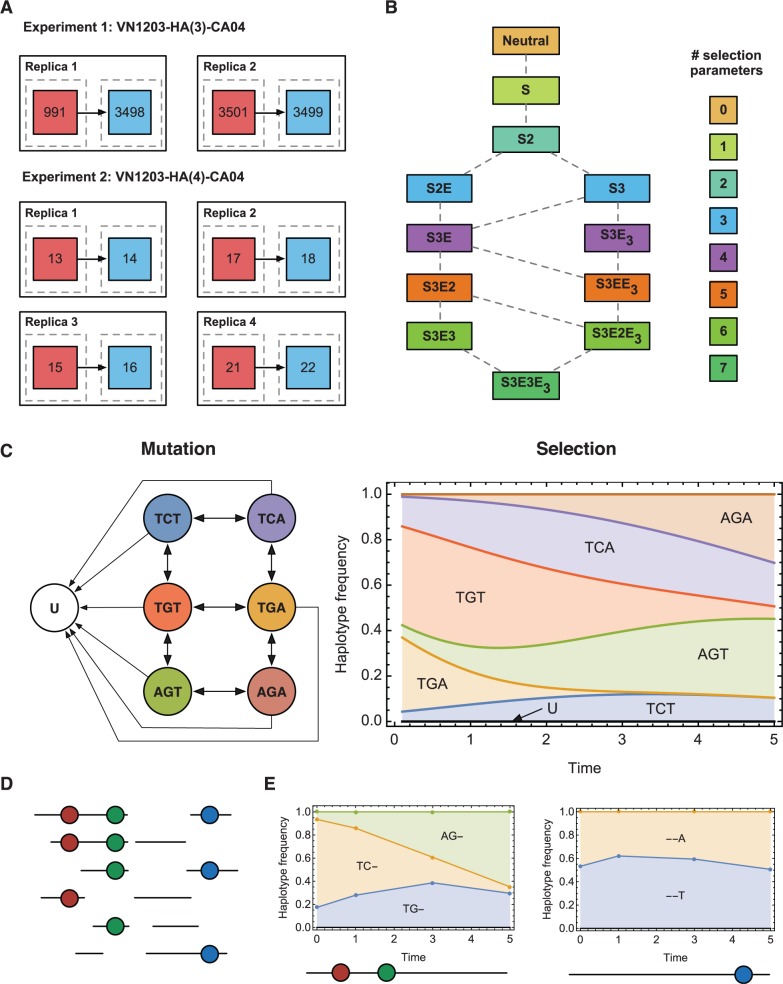
Overview of the method. (*A*) Original experimental setup. A large dose of virus was given to a ferret (red), contact (via airborne transmission) with a second ferret (blue) being initiated 1 day later. Two different viral strains were used; sequence data from two and four replicate experiments being described in each case. (*B*) Network of potential fitness landscape models for an example three-locus haplotype system. Here, S indicates an allele under constant selection, E indicates a two-locus epistatic interaction between alleles, and E_3_ indicates a three-locus epistatic interaction between alleles. Each model represents multiple potential fitness landscapes according to the choice of selected alleles and selection coefficients; model S encompasses all landscapes with an allele at any one locus under selection of any strength. (*C*) Dynamics in a single model. Mutation occurs between observed haplotypes differing by a single allele, and to a class U, representing unobserved haplotypes. Under selection, the frequencies of the different haplotypes change over time. (*D*) Paired-end sequence reads span either one or multiple alleles of interest in the system. Reads are partitioned according to the alleles they span. (*E*) Sets of reads describe partial haplotype frequencies, to which evolutionary models are fitted.

Data analyzed in this study were collected from a total of 12 animals. A total of six replicate experiments were performed using each virus; transmission was observed in two cases with the virus VN1203-HA(3)-CA04, and in four cases with the virus VN1203-HA(4)-CA04. Samples were collected from each animal every 2 days, beginning 1 day after innoculation, through nasal wash. For each sample from the animals between which transmission was observed, deep sequencing was conducted using an Illumina MiSeq platform for each of the HA, NA, and M1 genes (Wilker et al. 2013). A simplified overview of the experimental setup is shown in figure 1*A*.

Inference of fitness effects was conducted by means of a search over a hierarchical network of fitness models, beginning with a neutral model in which no selection was at work, and extending this to increasingly complex models by the addition of alleles under selection, and epistatic interactions between selected alleles (fig. 1*B*). Each model describes a space of possible fitness landscapes, characterized by the choice of alleles under selection, and the magnitude of selection applying to each. The evolution of the system given a fitness landscape was evaluated according to a model of cycles of mutation and selection acting upon the viral population, with both effects changing the frequencies of viral haplotypes over time (fig. 1*C*). Within a model, parameters describing the fitness landscape, and the initial state of the system, were optimized to generate a maximum-likelihood fit to the data, the complexity of the model being accounted for using the Bayesian Information Criterion (BIC) (Schwarz 1978). A model was accepted if there was strong evidence that it gave a better explanation of the data, accounting for model complexity, than any of the adjacent models in the network; less complex models were favored in the absence of such evidence.

In a first analysis, data were considered at the single-locus level, identifying loci at which significant changes in allele frequency were observed. Next, the sequence data were processed into sets of partial haplotype data. Individual paired-end reads span different subsets of loci of interest within the genome (fig. 1*D*), thus reporting “partial haplotypes” across different subsets of the loci of interest. For each subset, the partial haplotypes observed changed in frequency over time (fig. 1*E*). An underlying model describing the evolution of the underlying full haplotypes was learnt so as to simultaneously explain the changes of frequencies in each of these subsets. Considering the experimental data, we inferred a fitness landscape describing changes in frequencies observed across sets of animals, different viral populations having different initial genetic compositions, but being subject to a common underlying fitness landscape.

### Inferences from Simulated Data

**Fig. 2.**
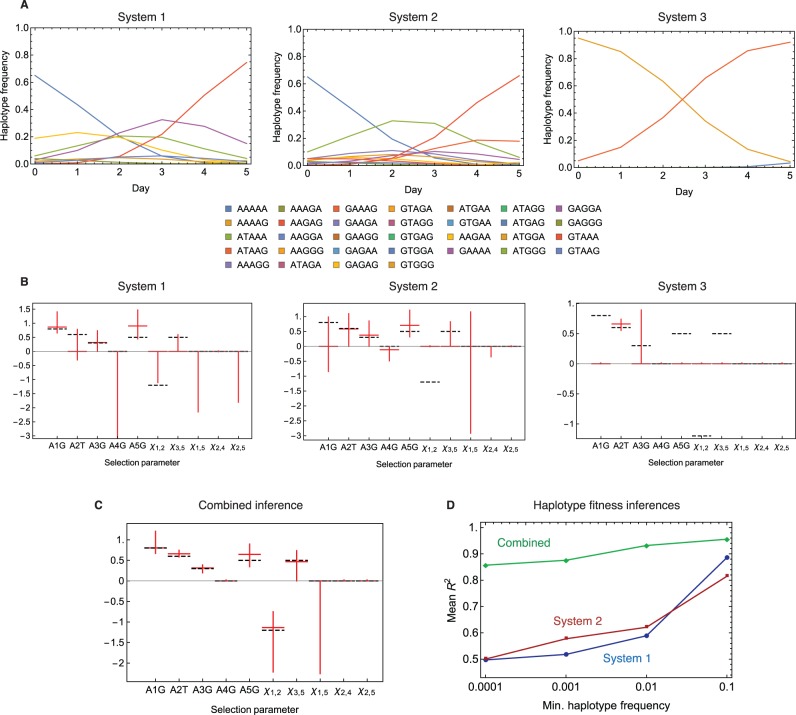
Inferences made from simulated data. (*A*) Full-haplotype frequencies were simulated using a model of mutation and selection with epistasis. Observations were recorded at times 0, 1, 3, and 5 for all one-, two-, and three-locus partial haplotypes. (*B*) Median inferred fitness parameters across ten independent sampling and inference calculations (red horizontal bars) compared with the true model values (horizontal black dotted lines). Vertical red bars show the range of inferred values across replicate simulations. (*C*) Median inferred parameters inferred from inferences combining data from each independent set of three simulated systems. (*D*) Mean correlations between real and inferred haplotype fitnesses for haplotypes inferred to exist at above a given haplotype frequency. A minimum of three haplotypes was required to generate a correlation.

Application of the method to simulated data showed the method to be effective in reconstructing a fitness landscape, despite the potential for variance in individual fitness parameters. Inferences were made from replicate samples taken from three populations, each evolving on a common fitness landscape (fig. 2*A*). Inferences derived from these data were generally accurate, with the limitation that the potential to infer a fitness landscape is restricted by the extent that the viral population explores that landscape (fig. 2*B*). For example, in system 3, the vast majority of the population was comprised only two haplotypes, AAAAG and ATAAG. Across the replica samples drawn from this system, the magnitude of selection was accurately inferred for the mutation A2T, which discriminates the haplotypes. However, fitness effects acting upon other variants were generally not identified; what was observed of the evolution of the system could be explained without further fitness parameters. In certain cases, an improvement in the inferred fitness landscape can arise through an incorrect allocation of fitness parameters. In system 1, the only haplotype surpassing 1% frequency with variants at the first two loci was the haplotype GTAAG. In one of ten replicates, negative epistasis between the variant alleles at these loci was correctly identified, but in other simulations negative epistasis was inferred to act either between the variant alleles at loci 1 and 5, or at the loci 2 and 5. All of these inferences improved the estimated fitness of the haplotype GTAAG, but not all were correct; haplotype fitnesses, rather than their composite parameters, are optimized by this approach.

Variance in the inferred fitness parameters occurred due to the finite sampling process. For example, in system 1, positive selection was always found for the variant A1G, but varied in magnitude from +0.7 to +1.4 (correct value +0.8). Noise also affected which parameters were identified in the selection model; selection was inferred to act upon the variant A2T in five of ten samples, but there was insufficient evidence to infer selection at this locus in the other five replicas. Increasing the depth of sampling improved the accuracy of the inferences that were made (supplementary fig. S1, Supplementary Material online).

A great improvement in the accuracy of the method was produced by performing an inference over samples from each of the three systems (fig. 2*C*). To the extent that different samples provide independent explorations of the fitness landscape by the viral population, substantially more data are available for inferences to be made. In the combined inference, all seven fitness parameters were inferred with good accuracy in eight of ten cases. In our analysis of the real data, we report inferences made across three and eight viral populations.

The maximum inferred frequency of a haplotype provided a good proxy for the accuracy with which its fitness was inferred (fig. 2*D*). In the combined inference, a good match to the correct haplotype fitnesses was produced across all haplotypes inferred to occur at a frequency of 10^−^^4^ or greater. In our analysis of the real data, we report inferences for haplotypes reaching an inferred frequency of 1% or greater.

Further simulations were performed to evaluate the vulnerability of the inference method to variation in the underlying mutation rate of the system. The correlation between the real and inferred fitness parameters was not substantially affected by an incorrect mutation rate (supplementary fig. S2, Supplementary Material online).

In common with other methods for inferring selection from time-resolved data, the range of selection coefficients that it is possible to measure depends on the relationship between the interval between samples and the magnitude of selection (Illingworth and Mustonen 2012; Feder et al. 2014). If selection is too weak, allele frequencies will not change sufficiently within the recorded interval for selection to be identified. If selection is too strong relative to the interval of sampling, fixation can occur too quickly for selection to be quantified.

### Inferences from Experiment 1

Application of the method to the experimental data provided strong evidence for selection acting at multiple alleles in each viral population. Between the different experiments, alleles at different sets of loci were identified as being under selection, compatible with differences in the low-frequency standing variation contained within the two initial populations. However, where selection was inferred at the same locus in each experiment, similar fitness parameters were obtained.

**Fig. 3.**
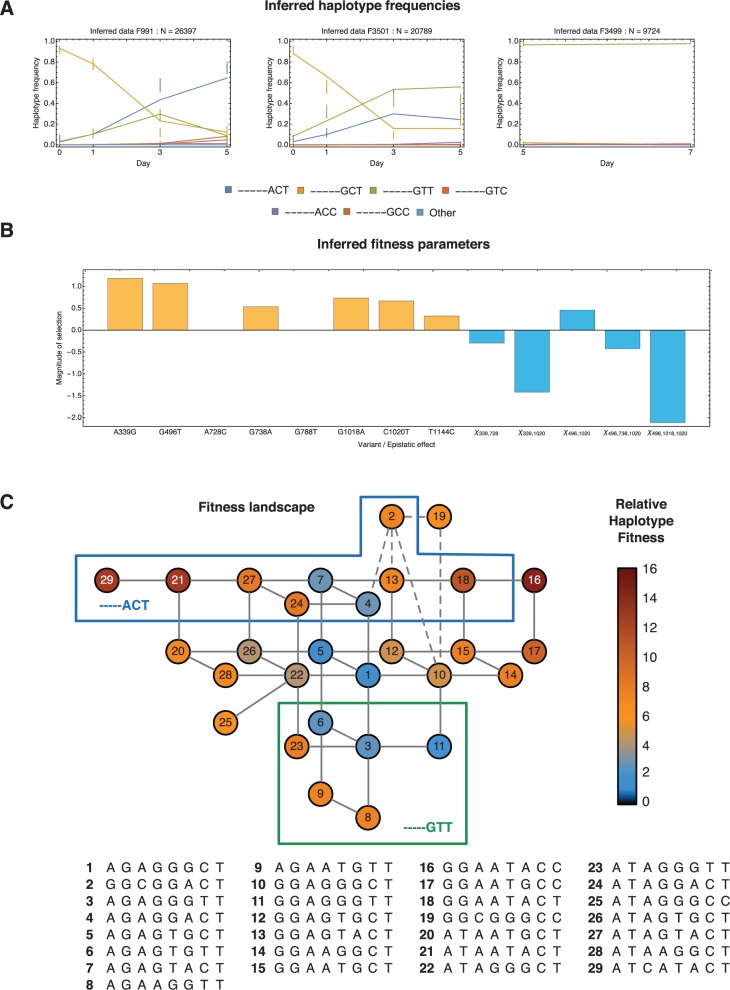
Inferences made for experiment 1. (*A*) Observed and inferred partial haplotype frequencies. Haplotype frequencies from reads spanning the final three loci of interest are shown as vertical bars. Bars are centered on the observed frequency and extend one standard deviation either side of this, according to the inferred model of noise in the data. Inferred haplotype frequencies are shown as solid lines. (*B*) Inferred selection coefficients (yellow) and epistatic interactions (blue) given the default mutation rate *μ* = 10^−5^. (*C*) Inferred fitness landscape. Solid lines between numbered haplotypes indicate single mutations. Dotted lines indicate haplotypes separated by two mutations. Inferred fitness is indicated by color. Only haplotypes that were inferred to comprise 1% or more of the population in some animal at some time point are shown.

Within the data from experiment 1, evidence for significant changes in allele frequency was found at eight loci in the HA genome. Considering changes in partial haplotype frequencies across these loci, selection was inferred to act upon six of these, on the variants A339G, G496T, G738A, G1018A, C1020T, and T1144C. Combining these inferences into a fitness landscape provided intuition regarding the observed patterns of evolution. For example, in experiment 1, two different outcomes were observed in the ferrets 991 and 3501. In the former, haplotypes with the alleles ACT at loci 1018, 1020, and 1144 became dominant by the end of the period of infection, whereas in the latter haplotypes with the alleles GTT at these loci were the most prevalent by the end of the infection (fig. 3*A*). The fitness landscape suggests that the difference of outcomes may be explained by the existence of multiple potential pathways of evolution. From the initial consensus haplotype, labeled 1, a gain in fitness can be achieved by moving through the fitness landscape toward either those haplotypes characterized by ACT or those haplotypes with the alleles GTT (fig. 3*B*). Small, stochastically induced differences in the initial viral population (infections were initiated with large doses of the same viral stock) can push the early development of the viral population in one direction or another, leading to substantial changes in outcome.

Results from experiment 1 highlighted the importance of accounting for linkage disequilibrium in discerning selection from viral sequence data. In each of the replicate animals 991 and 3501, a substantial increase in the frequency of the G788T mutation was observed from an initial 3% of the input population to 73% and 24%, respectively, at the final time of sequencing. However, the sequence data collectively did not support the inference of positive selection at this locus; a model of hitchhiking with other selected alleles gave a better explanation (when model complexity was accounted for) of the observed frequency changes.

### Inferences from Experiment 2

**Fig. 4.**
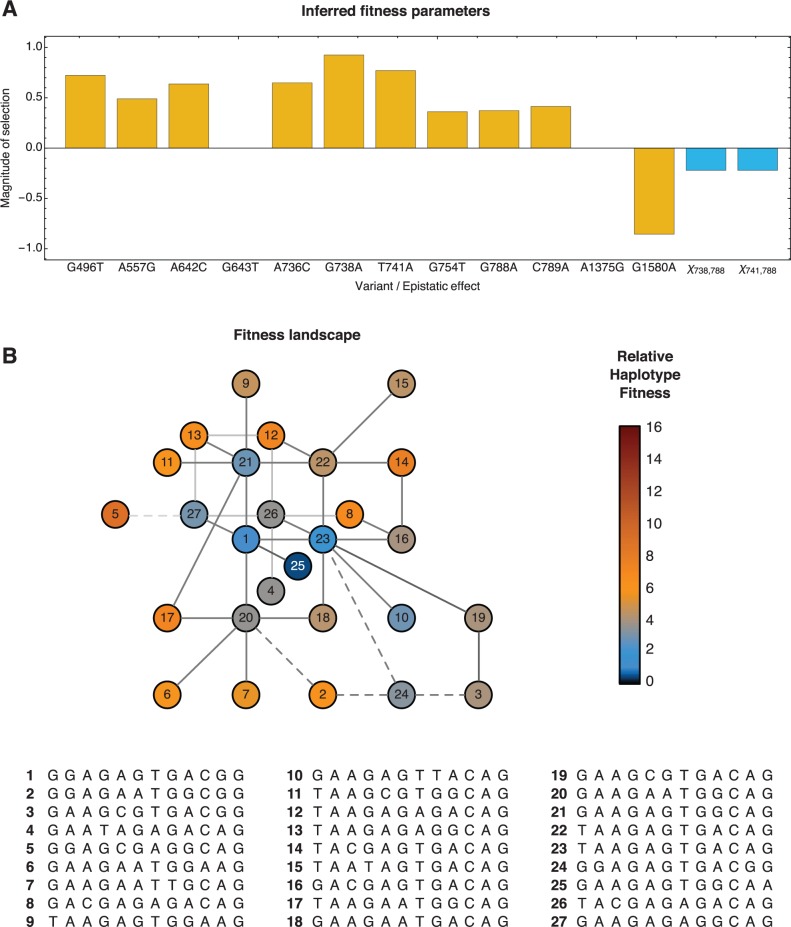
Selection coefficients inferred for experiment 2. (*A*) Inferred selection coefficients (yellow) and epistatic interactions (blue) given the default mutation rate *μ* = 10^−5^. (*B*) Inferred fitness landscape with the default mutation rate. Haplotypes reaching an inferred frequency greater than 1% are shown. Haplotype fitnesses are described relative to the initial consensus haplotype, which has the label 1. Lines indicate haplotypes separated by single nucleotide mutations.

Data from experiment 2 expanded upon the finding of multiple pathways toward adaptation. The input viral stock used in this case differed only by a single intentional mutation from that used in experiment 1, albeit very different patterns of lower-frequency variation were observed across other genetic loci (Wilker et al. 2013). This variation led to polymorphisms being identified at an overlapping, but substantially different, set of loci. Evidence for significant changes in frequency was found at 12 loci in the HA genome, with selection being inferred to act upon the 10 variants G496T, A557T, A642C, A736C, G738A, T741A, G754T, G788A, C789A, and G1580A (fig. 4). Further details of parameters inferred from each experiment are contained in supplementary tables S1 and S2, Supplementary Material online; inferred parameters from individual animals are shown in supplementary figures S3 and S4, Supplementary Material online.

Differences between experiments in the loci at which selection was inferred are consistent with differences in the initial composition of the each viral population; variants at higher initial frequencies are far more likely to be picked up by selection within the timescale of a single infection. Where identical mutations were identified in both experiments, similar fitness parameters were inferred (supplementary fig. S5, Supplementary Material online).

### Inference of Epistatic Effects

Strong evidence for epistatic effects was found in the data from both experiments. Of particular interest is the inference of strong negative epistasis was inferred between the variant alleles at the loci 339 and 1020; the A339G and C1020T mutations were each inferred to be independently under positive selection, but lose a great deal of the benefit they provide in the presence of the other mutation. Due to the importance of structural stability for the influenza virus, epistatic effects are likely to be prevalent in the viral fitness landscape (Wylie and Shakhnovich 2011). Epistatic fitness effects have previously been identified in influenza by phylogenetic analyses of HA and NA sequence data (Kryazhimskiy et al. 2011), and from the introduction of mutations into reconstructed historical sequences of the nucleoprotein gene (Gong et al. 2013); cases where mutations have background-dependent effects upon phenotype have been listed elsewhere (Lipsitch and Galvani 2014). Our results suggest that epistatic fitness interactions play a significant role in the short-term, within-host evolution of the virus.

### Functional Consequences of Selected Mutations

The identification of selection acting across alleles at multiple loci is consistent with previous reports of many of these variants having functional consequences. For example, the A736C, G738A, and T741A variants (which translate to the protein substitutions K224N, G225E, and Q226L) have each been associated with mammalian host receptor specificity, either in H5N1 directly or in other influenza viruses (Matrosovich et al. 2000; Wilker et al. 2013). The substitution T741A, identified as being under positive selection, was one of the mutations introduced to create the VN1203-HA(3)-CA04 virus, and was implicated in causing the shift from Siaα2,3Gal to Siaα2,6Gal recognition (Imai et al. 2012). The A736C substitution is the inverse of another introduced mutation, again associated with Siaα2,6Gal recognition.

Among the other selected variants, the G496T mutation (K144N) has been identified in human H3N2 viruses (Eshaghi et al. 2013), being associated with immune escape through glycosylation (Das et al. 2011). The A557G (K165E) variant has been identified as increasing receptor binding avidity in the H1 HA (Hensley et al. 2009). The C1020T (T318I) mutation in the virus VN1203-HA(3)-CA04 is the sole intentional difference between the viruses used in the two experiments, and has been associated with increased HA stability, and improved transmissibility. While being fixed in the VN1203-HA(4)-CA04 virus, we infer it to be under positive selection in the former viral population, albeit that this effect is severely weakened by the presence of A339G.

Of the remaining substitutions, the mutations G1018A and T1144C are synonymous in character; selection for synonymous mutations has previously been identified in multiple RNA viruses, including influenza (Kryazhimskiy et al. 2008; Cuevas et al. 2011; Illingworth et al. 2014). The mutation G788A (A242T) has been noted in previous studies of H5N1 (Imai et al. 2012), and is relatively close to the receptor binding site in the HA trimer structure, albeit across the boundary between two monomers (supplementary fig. S6, Supplementary Material online); C789A causes the A242E substation in the same amino position. The mutations A339G, A642C, and G754T cause the D101G, K193T, and M230I substitutions close to the top of the HA protein, whereas G1580A causes an E to K mutation at the base of the HA2 protein.

### Rapid Increase in Population Mean Fitness

**Fig. 5.**
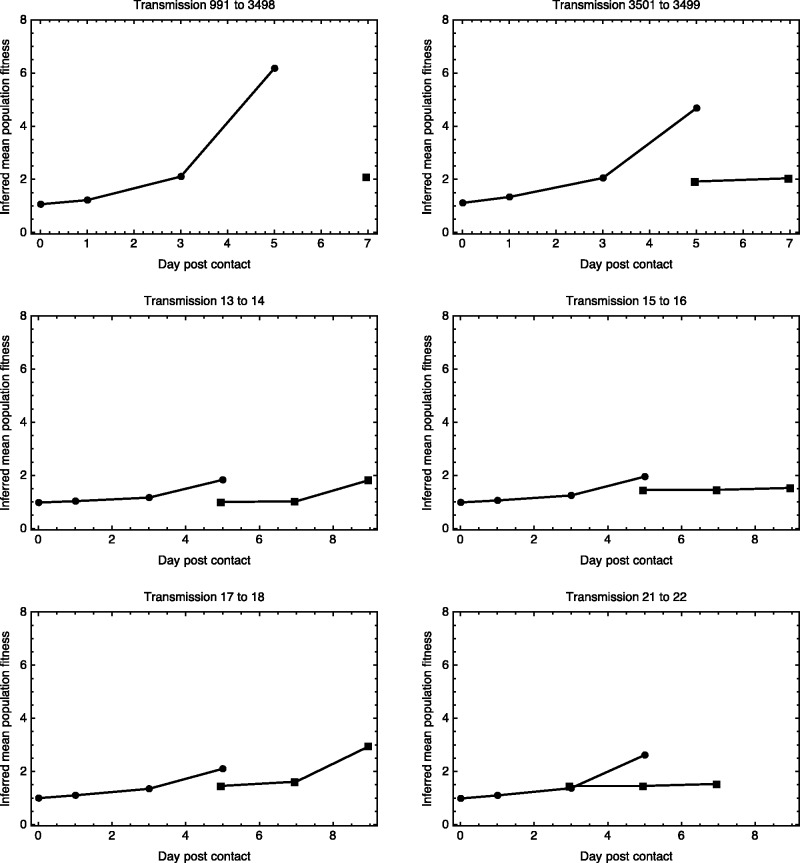
Within-host adaptation of the virus. Inferred values of the mean fitness of the viral population in index (circles) and contact ferrets (squares). The mean fitness of the population increases, with adaptation proceeding at an increasing rate.

Inferred changes in the mean fitness of the viral population over time suggest that the HA gene of the reassortant virus is initially very poorly adapted to within-host growth, with the process of within-host evolution being severely limited by the length of a single infection. The inferred mean fitness of the viral population increased substantially during the length of the infection in each population (fig. 5). In addition, the rate at which the population gained in fitness itself increased over time. This suggests that the viral population remains far from approaching a peak in the fitness landscape; were this the case, the rate of adaptation would tail off with time. Given that the greatest gain in inferred fitness occurred in the final time interval, either a much longer period of infection or multiple consecutive passages of the viral population would likely be required if the population were to approach peak fitness.

In most cases, the mean fitness of the viral population was reduced upon transmission; the fittest haplotype for within-host growth in the index ferret was not transmitted. The lack of diversity in the posttransmission populations has been noted elsewhere (Wilker et al. 2013). Such a bottleneck explains the low rates of within-host adaptation inferred here posttransmission. Given a lack of diversity, more beneficial haplotypes have to be generated through mutation before experiencing selection, leading to a delay in viral adaptation. In both the index and contact ferrets the limited timespan of the infection limited the extent of adaptation that was achieved.

### Conservation of Results under Variance in the Model Mutation Rate

Study of the vulnerability of the results to changes in the mutation rate used for inference was conducted using data from experiment 1. Under a 100-fold variance in the mutation rate used in the model, including variance in the transition–transversion ratio, the general topology of the inferred fitness landscape was preserved (supplementary fig. S7, Supplementary Material online). Of the parameters described above, all of the alleles inferred to be under selection, and the finding of negative epistasis between the variants A339G and C1020T, were preserved across all tested mutational models. Details of inferred parameters under different mutation rates are shown in supplementary table S3, Supplementary Material online, whereas inferred fitness landscapes are shown in supplementary figure S8, Supplementary Material online. Greater knowledge of the true mutational process would provide greater precision in the inferred magnitudes of selection at work, and a greater resolution in describing the fitness landscape.

## Discussion

We have developed a method for inferring the within-host fitness landscape of influenza based upon error-prone short-read sequence data. Our method uses the partial description of linkage disequilibrium between alleles available from paired-end reads to reconstruct a set of underlying viral haplotypes, using these as the basis for an evolutionary model. A search, conducted over a hierarchical set of classes of fitness landscapes, gives the model of selection best fitting the observed sequence data. The resulting inference is not comprehensive, in that fitness effects additional to those described almost certainly apply to the virus; only those effects producing significant changes in allele frequencies can be identified. The fitnesses of haplotypes that do not appear at substantial frequencies in the population, whether due to purifying selection or distance in sequence space from the initial population, cannot be accurately inferred. Nevertheless, on the basis of a conservative statistical framework, and under the given modeling assumptions, our approach captures the main components of the fitness landscape underlying changes in the composition of the viral population, as evidenced from the sequence data.

Aspects of our method are potentially broadly applicable to short-read viral data. In this study, we have applied our method to data from an evolutionary experiment describing the transmission of a reassortant A/H5N1 influenza virus between ferrets. In these experiments, we inferred selection for within-host growth to be acting upon multiple mutations, including those responsible for receptor binding specificity, immune escape, and protein stability.

Attention has been given to the question of what might be learnt from evolutionary experiments conducted using influenza viruses (Casadevall et al. 2014); our approach increases the amount of information that may be derived from sequence data, providing, in this case, a quantitative understanding of potential evolutionary mechanisms by which an increased transmissibility of the H5N1 virus might emerge. As has been considered in detail elsewhere, within- and between-host fitness effects are both of importance for the emergence of a novel pathogen (Coombs et al. 2007); our inferences at the within-host level would need to be combined with knowledge from other sources. For example, the C1020T mutation, associated with increased transmissibility (Imai et al. 2012), appears to be under positive, though context-dependent, within-host selection in ferrets.

Our results highlight potential limitations in the use of evolutionary experiments to predict the behavior of natural influenza populations. It is possible, even where changes in the phenotype of a population are deterministic in nature, for the genetic nature of that adaptation to be highly stochastic (Kryazhimskiy et al. 2014). In the data here, we identify multiple different genetic pathways through which the influenza virus can evolve following a host-switch event. Both within and across the two experiments, the precise pathway taken was dependent upon small differences in the population of viruses that established the infection. Even where a large population of virus was used to initiate replicate experiments, variation in the outcome was observed, representing different paths to adaptation. Although the number of such paths is clearly finite, further data might be required to predict the evolution of another, identical replicate. Given experiments with different genetic starting points, further pathways would likely be discovered; the complete fitness landscape is far greater in scope than the fragments inferred here. It is therefore likely that substantially more data would be required to predict which route to increased transmissibility the evolution of the H5N1 virus might take in the wild, if such evolution were to occur. Clinically relevant predictions of the evolution of HIV have been based upon large data sets of evolutionary pathways (Beerenwinkel et al. 2005); similarly extensive data might be required in this case.

Our results pose similar challenges for the task of viral genetic surveillance. Although genotype–phenotype predictions offer some promise for evaluating the threat of potential pandemics (Russell et al. 2014), the potential for phenotypic change through multiple genetic routes and the presence of strong epistatic effects together highlight the fact that simplistic approaches to this task are likely to fail. If an observed strain did not directly possess the phenotypic properties required to cause a pandemic, assessing the risk posed by its future evolution would require a comprehensive understanding of the potential routes the strain could take toward adaptation. Such an understanding would require a characterization of regions of the fitness landscape lying far beyond those explored within a single evolutionary experiment.

## Materials and Methods

The within-host evolution of the viral population was described through a simple evolutionary model accounting for the effects of mutation and selection upon the population over time (Illingworth et al. 2014). In our model, we consider the viral population at the level of haplotypes, each encompassing the set of sequences with specific alleles present at a defined series of genomic loci. Our model describes changes in haplotype frequencies during the course of an infection; if the haplotypes differ in fitness, the frequencies of more beneficial haplotypes will increase over time. Different models were evaluated against observations of sequence data from the infection; a likelihood model was used to identify the most likely role for selection acting upon the virus. In constructing our model, a deliberately conservative approach was taken, both in estimating the extent of noise in the data and in demanding strong statistical evidence before inferring the presence of selection on a genetic variant.

### Within-Host Evolutionary Model

We describe the viral population as a vector *q* of haplotype frequencies, such that the frequency of the viral haplotype ***a ***at time *t_k_* is given by the value *q****^a^***(*t_k_*). A haplotype is here defined as a short sequence of nucleotides spanning a set of (not necessarily adjacent) loci within a single viral gene.

Over time we model the population as evolving through a process of repeated cycles of intracellular replication and release of viruses, followed by the infection of new host cells. Within cells, we assume that each new virus undergoes two error-prone strand-copying events before being released from the cell. Outside of the cell, selection acts upon the viral population before new cells are infected. We thus model the evolution of the population using the equation

$$q(t_{k+1})=S(M^{2}(q(t_{k}))),$$

where the matrix *M* describes the effect of mutation on the population, and the function *S* describes the effect of selection. Within-host reassortment between genes was assumed to be rapid (Marshall et al. 2013). Time points in our model were spaced at 12-h intervals, such that mutation occurs at a frequency approximating the time for a round of intracellular growth (Baccam et al. 2006). The effects of selection and mutation were characterized as follows.

### Mutation

The matrix *M* describes the probability of mutation from one haplotype to another in a single strand-copying event. Where haplotypes each have *L* loci, if all potential haplotypes are considered, there are *4^2^^L^* such transitions, such that *M* becomes large. We therefore implemented an approximate mutation matrix of the form

$$M=\left[\begin{array}{cc} E & 0\\ T & 1 \end{array}\right],$$

where E is a matrix, T is a row vector, and 0 is a column vector. In this model, potential haplotypes are divided according to whether or not they were inferred, on the basis of the short read data, to have been observed in the viral population (see below). The matrix *E* describes mutation between observed haplotypes, having elements defined by:

$$\begin{array}{ll} E_{ij}=1-L\mu & i=j\\ =\mu /3 & i\neq j,\;H(i,j)=1\\ =0 & i\neq j,\;H(i,j)>1 \end{array}$$

where *μ *is the rate of mutation from one specific nucleotide to another at a single locus, *L* is the number of loci in each haplotype, and *H*(*i, j*) is the Hamming distance between the haplotype sequences *i* and *j*. The value of *μ *was here estimated to be approximately 10^−^^5^ (Sanjuan et al. 2010), although the potential variance in this measure is high (Acevedo et al. 2014). Certain calculations were therefore repeated with mutation rates in a 100-fold range around this, including variance in the transition–transversion ratio. Mutations involving multiple nucleotide changes occur at a rate of order *μ*^2^; as *μ *in each case is small these were neglected.

The vector *T* describes mutation from the observed haplotypes to nonobserved haplotypes, and is composed of the elements *μt_i_*/3, where *t_i_* is the number of the *3L* mutations out of the haplotype *i* that do not result in another observed haplotype.

Finally, the vector 0 is a column vector composed of zeroes; mutations from nonobserved to observed haplotypes were neglected. In the viral population nonobserved haplotypes are substantially greater in number than observed haplotypes, but their total frequency as a fraction of the population is low. As such, the total rate of mutation out of nonobserved haplotypes will be low, and the majority of these mutations will be to other, nonobserved haplotypes.

### Selection

Fitness values were assigned to all haplotypes on the basis of the alleles, and combination of alleles within each haplotype, and relative to the “wild-type” haplotype, which was defined as the haplotype with the initial consensus allele at each position. Changes in haplotype frequencies were modeled according to the equation:

$$S(q^{a}(t_{k}))=\frac{q^{a}(t_{k})\text{exp}(\sigma_{a})}{\sum_{b}q^{b}(t_{k})\text{exp}(\sigma_{{}^{b}})},$$

where *σ*_a_ is a sum of fitness parameters describing the selective benefit or disadvantage of the specific alleles, or combinations of alleles in the case of epistasis, contained within the haplotype *a*; in the simplest case, under the neutral model, *σ_a_* = 0 for all *a*. The Darwinian fitness of the haplotype *a,* reported in the fitness landscapes above, is given by

$$f_{a}={\text{e}}^{\sigma_{a}}.$$

The mean fitness of the population at the time *t_k_* is given by

$$\bar{f}(t_{k})=\sum_{a}f_{a}q^{a}(t_{k}).$$

Haplotype fitness is assumed to remain constant over time, giving a well-defined fitness landscape (Mustonen and Lässig 2009). The values *σ_a_* and the initial haplotype frequencies *q^a^*(*t_0_*) together define the evolution of the system. A likelihood model was used to compare inferred patterns of evolution with the observed sequence data.

### Collection of Data

Data from a previous study, which described the evolution of reassortant H5N1 viruses in ferrets, were analyzed (Wilker et al. 2013). In the experiment described by this study, ferrets were inoculated with a large dose of one of two reassortant viruses, and were housed in close proximity to naïve ferrets, preventing physical contact between animals, but allowing for airborne transmission of virus. In six of the experiments conducted, airborne transmission between animals was observed; samples of virus were collected at multiple times from both the donor and the recipient ferrets in each case and sequenced (Wilker et al. 2013).

### Processing of Sequence Data

The short sequence reads collected from next-generation sequencing do not necessarily report haplotypes spanning all of the loci of interest in the viral genome. As such, the observed sequence data were processed into partial haplotype data.

An initial alignment of sequence reads was conducted using SMALT v0.7.4.4, aligning reads of the HA gene to the A/Vietnam/1203/2004 reference sequence, and reads of other genes to the A/California/04/2009 reference sequence. A more detailed processing of reads was then conducted using the within-house application SAMFIRE. Following an approach similar to that of the QUASR software package (Watson et al. 2013), reads with median quality less than 30 were trimmed from either end until either a median score of 30 was achieved or until the sequence was reduced to having fewer than 30 nucleotides with read quality greater than or equal to 30. Where sufficient quality was achieved by trimming from more than one end, the longest resulting sequence was retained. Reads were then individually aligned to the appropriate reference sequence using the MUSCLE software package (Edgar 2004). Primer sequence beyond either end of the reference sequence was removed, as were reads with less than 95% sequence identity with the reference, or in which insertion or deletion events were inferred. From each remaining read, individual nucleotides with read quality less than 30 were removed, each such nucleotide being recorded as a gap in the sequence. Sequences with at least 30 high-quality nucleotides remaining were kept for further analysis. The resulting reads were used to calculate single-locus measures $n_{i}^{a}$ of the numbers of observations of each nucleotide *a* at each locus *i* in the viral genome.

### Estimation of Noise in the Sequence Data

To generate a likelihood model comparing each inferred model to the collected observations, an estimate was made of the extent of noise in the data. We note that differences between true and observed allele frequencies could arise for many different reasons. The viral samples, collected through nasal wash, may not be fully representative of the viral population within an animal. Processing of these samples through PCR may distort the composition of the sampled population. Despite substantial processing, sequencing errors are likely to remain in the data. Further, the actual observations represent a finite sample of the population. The effect of a noisy, finite sample was modeled using a Dirichlet multinomial likelihood; we here define

$$L^{D}(N,C,\text{q},\text{n})=\log\frac{\Gamma (N+1)}{\prod_{a}\Gamma (n^{a}+1)}\frac{\Gamma (\sum_{a}Cq^{a})}{\Gamma (\sum_{a}n^{a}+Cq^{a})}\prod_{a}\frac{\Gamma (n^{a}+Cq^{a})}{\Gamma (Cq^{a})},$$

where the vector ***n**** = {n^a^}* describes the number of observations of type *a*, *N* is the total number of observations, the vector ***q**** = {q^a^}* describes the underlying frequency of individuals of type *a*, and the parameter *C* characterizes the extent of overdispersion; here, lower values of *C* represent greater overdispersion relative to a standard multinomial model.

The parameter C was estimated by fitting a model of neutral evolution to data from “potentially neutral” systems in which relatively little change in allele frequency was observed. Conservatively, populations in which all allele frequencies changed by an average of less than 5% per day were identified for this purpose; this included data describing the MP gene from all animals, describing the NA gene from all except ferret 22, and describing the HA gene from ferrets 16, 22, and 3499 (supplementary fig. S9, Supplementary Material online). Considering the data at the single nucleotide level, nucleotide frequencies were collected for each of these populations across all loci for which a minority allele frequency of at least 1% was observed. At each locus *i*, the mean frequency of each nucleotide was calculated by summing observations across the time points *t_k_*:

$${\bar{q}}_{i}^{a}=\frac{\sum_{k}n_{i}^{a}(t_{k})}{\sum_{k}n_{i}(t_{k})},$$

where *n_i_*(*t_k_*) is the total number of observations at time *t_k_*. The likelihood of the observation at time *t_k_* was then given by the likelihood

$$L_{i}^{d}(t_{k},C)=L^{D}(N_{i}(t_{k}),C,{\bar{q}}_{i},n_{i}(t_{k})),$$

where *d* denotes the animal from which the data were collected, ***n_i_*(*t_k_*)** is the vector of nucleotide frequencies at the locus *i* and *N_i_*(*t_k_*) is the total number of observations of the allele *i* at *t_k_*. Summing these likelihoods over all trajectories from all of the populations considered gave the overall likelihood

$$L(C)=\sum_{d,i,k}L_{i}^{d}(t_{k},C).$$

Optimizing this likelihood with respect to *C* gave a maximum-likelihood estimate for the extent of overdispersion in the single-locus data, *C_sl_*.

### Identifying Potentially Nonneutral Loci

Following the approach of an earlier analysis of within-host influenza data (Illingworth et al. 2014), we next identified potentially nonneutral loci, defined as loci at which significantly nonneutral changes in allele frequency were observed. For each population, loci at which minority alleles were observed at a frequency of at least 1% were considered. The within-host evolutionary model described above was optimized to fit the allele frequencies at each locus, comparing models of neutral evolution and selection acting upon the minority allele. The likelihood of each model *m* was given by

$$L_{m}=\sum_{k}L^{D}(N_{i}(t_{k}),C_{sl},q_{i}(t_{k}),n_{i}(t_{k})),$$

where ***q_i_*(*t_k_*)** is the vector of inferred nucleotide frequencies from the optimized model at time t_k_. Models were compared using the BIC

$${\text{BIC}}_{m}=-2L_{m}+k_{m}\log N,$$

where *k_m_* is the number of selection parameters in the model *m* and *N* is the total number of observations of nucleotides at the locus considered. A lower BIC score indicates a better fit to the data given the complexity of the model; potentially nonneutral loci were identified at those for which the model of selection gave a lower BIC than did the neutral model for at least one animal in the experiment.

### Deriving Partial Haplotype Data

Having identified loci of potential interest, sequence data were processed into haplotypes spanning subsets of the potentially nonneutral loci. First, high-quality paired-end reads were joined together into pairs, consisting of single, longer sequences. Where the reads covered disjoint parts of the sequence, alleles in between the reads were recorded as gaps; that is, as loci for which no information was provided by the pair.

Next, denoting the set of all pairs collected from a single animal at a single time as *P*, we note that *P* can be divided into distinct subsets. Supposing there to be *L* potentially nonneutral loci, the set $P_{1}^{L}$ was defined as the set of pairs in P that reported a nongap nucleotide at every one of the L loci. Next, the sets $P_{1}^{L-1}$ and $P_{2}^{L-1}$ were defined as the sets of pairs not in $P_{1}^{L}$ that reported nongap nucleotides at the two sets of *L −* *1* consecutive potentially nonneutral loci, {*1*, … , *L−1*}, and {*2*, … , *L*}. Continuing in this manner we defined sets of paired reads reporting nongap nucleotides at fewer and fewer consecutive loci, the final sets $P_{1}^{1}$ to $P_{L}^{1}$ consisting of pairs that reported a single nongap nucleotide at any one of the potentially nonneutral loci. Each subset $P_{1}^{l}$ of P thus reports a set of *l*-locus partial haplotypes across a set of consecutive potentially nonneutral loci.

**Fig. 6.**
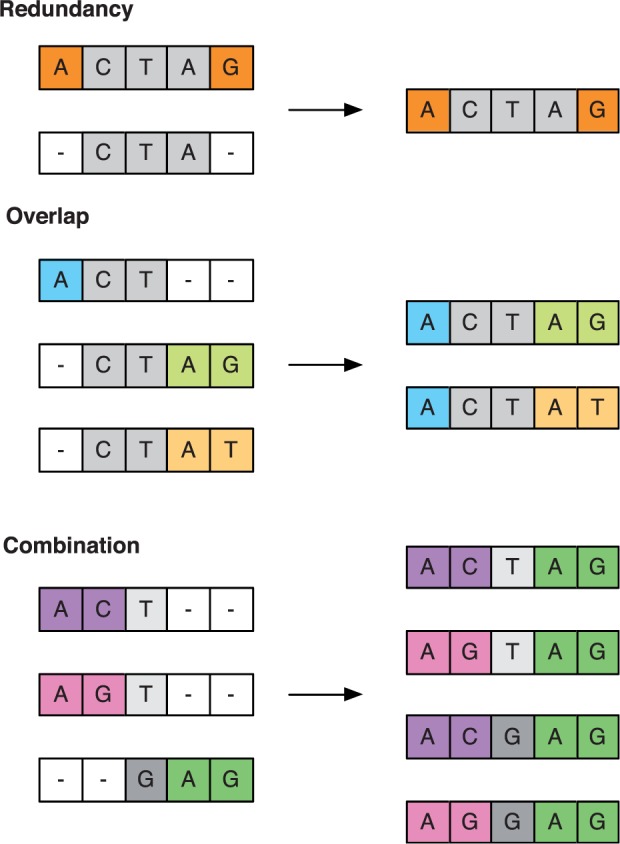
Inference of haplotypes from partial haplotype observations. Three processes were used to infer underlying haplotypes. First, redundant partial haplotypes were removed from consideration. Second, overlapping partial haplotypes were joined into sets of longer haplotypes. Having repeated these two steps, all potential combinations of remaining partial haplotypes were generated.

Data from the partial haplotypes were used to reconstruct a minimal set H of full L-locus haplotypes from which the partial haplotypes could potentially have been emitted. To exclude rarely observed haplotypes, partial haplotypes were temporarily excluded if they had been observed on ten or fewer occasions in a subset $P_{i}^{l}$, or at a frequency of less than 1% within their subset. The remaining partial haplotypes were then combined by the application of three rules, as illustrated in figure 6:

#### Redundancy

We say that the partial haplotype *a_1_* is contained within the partial haplotype *a_2_* if *a_1_* reports identical alleles to *a_2_* at a subset of the alleles spanned by the haplotype *a_2_*. If a partial haplotype *a_1_* was contained within another partial haplotype *a_2_*, then *a_1_* could potentially have been emitted from the same *L*-locus haplotype as *a_2_*. As such, the partial haplotype *a_1_* was removed from consideration.

#### Overlap

Where partial haplotypes *a_1_* and *a_2_* spanned different loci, but shared a subset of their loci, and where *a_1_* and *a_2_* reported identical alleles at these common loci, both *a_1_* and *a_2_* could have been emitted by a common *L*-locus haplotype. A new haplotype combining *a_1_* and *a_2_* was created.

Having repeated these rules until no further change was produced in the haplotypes, some haplotypes of length less than *L* could potentially remain. In this case, a third rule was applied, generating combinations of haplotypes.

#### Combination

Where a partial haplotype *a* spanned a subset *l *of the *L* loci, all of the remaining haplotypes were used to generate the set of all distinct haplotypes spanning the complementary subset of all loci not in *l*. Every combination of the haplotype *a* with a partial haplotype in this complementary subset was generated and added to the set of haplotypes. This step allows the reconstruction of potential haplotypes even in the case where no explicit linkage data are available.

Next, partial haplotype reads were checked for consistency with the inferred full haplotypes, filtering out reads that could not have been emitted from one of these haplotypes. Across the two experiments, more than 99.5% of partial haplotype reads were consistent with the *L*-locus haplotypes inferred for the HA gene by the process above.

Application of the above method to data from simulations suggested it to be efficient in identifying low-frequency haplotypes given the quantity of data available in this case (supplementary fig. S10, Supplementary Material online).

### Likelihoods in the Full Evolutionary Model

The haplotypes in the set *H* were used to propagate the evolutionary model, with the likelihood of a specific model being calculated in terms of the partial haplotype observations. We note that each set of partial haplotype observations is independent from every other, as paired reads are assigned to at most one, well-defined set. The overall likelihood of the model is thus given by

$$L_{m}=\sum_{p}\sum_{k}L^{d}(N_{p}(t_{k}),C_{ph},q_{p}(t_{k}),n_{p}(t_{k})),$$

where *N_p_*(*t_k_*) is the number of observed partial haplotypes in each subset *p* of *P* at time *t_k_*, ***q_p_*(*t_k_*)** and ***n_p_*(*t_k_*)** are the vectors of inferred frequencies and observed numbers of partial haplotypes in *p* at time *t_k_*, and *C_ph_* is the appropriate level of overdispersion in a calculation utilizing the partial haplotype data. Inferred frequencies of partial haplotypes were calculated from the inferred frequencies of haplotypes in *H* calculated in the model; haplotypes not in *H* were considered as a single, additional haplotype. Again, a calculation of BIC was used for discrimination between models. Following a conservative approach to inferring selection at a given locus, a model with an additional parameter was only accepted if it brought about an improvement in the BIC score of at least 10, representing strong evidence in favor of the more complex model (Kass and Raftery 1995).

### Estimation of Noise in the Partial Haplotype Data

Calculation of *C_ph_* was carried out in a similar manner to the calculation of the extent of noise in the single-locus data. We note that, as the data are in this case being processed in a different manner, the resulting amount of noise may be different, requiring an independent estimate. For each of the identified potentially neutral systems, sets of partial haplotypes $P_{i}^{l}$ were calculated across all loci for which a minor allele frequency of at least 1% was observed. For each set of partial haplotypes, the mean frequency of each haplotype **a** was calculated as a proportion of the total number of observed haplotypes across all time-points in an animal, approximating the maximum-likelihood solution in the case of neutrality.

$${\bar{q}}_{P_{i}^{l}}^{a}=\frac{\sum_{k}n_{P_{i}^{l}}^{a}(t_{k})}{\sum_{k}n_{P_{i}^{l}}(t_{k})}.$$

The likelihood of the observation at time *t_k_* was thus given by

$$L_{P_{i}^{l}}^{d}(t_{k},C_{\mathrm{ph}})=L^{D}(N_{P_{i}^{l}}(t_{k}),C_{\mathrm{ph}},{\bar{q}}_{P_{\text{i}}^{\text{l}}},n_{P_{\text{i}}^{\text{l}}}(t_{k})).$$

Summing these likelihoods over all sets of partial haplotypes from all times and in all of the populations considered gave the overall likelihood function

$$L(C)=\sum_{d,l,i,k}L_{P_{i}^{l}}^{d}(t_{k},C_{\mathrm{ph}}).$$

To avoid overfitting parameters to the data, a conservative estimate of noise was calculated. Having identified a maximum-likelihood estimate for *C*, a region of the likelihood distribution was identified within which the likelihood was no more than 5 units lower than the maximum. The lower bound of this region was used in subsequent calculations. Based upon the likelihood surface, this represents a 99.7% lower bound for the distribution.

### Generation of Fitness Landscapes

Data from all of the animals within each experiment were used to generate each fitness landscape. Assuming that selection acts in a similar manner upon the viral population within each animal within each experiment, selection coefficients were inferred jointly across the different populations, allowing for differences in the initial haplotype frequencies within each animal. An optimization procedure was used to identify maximum-likelihood parameters for each model in question.

### Generation of Simulated Data

Five-locus haplotypes were generated, and assigned initial frequencies and selection coefficients. The system was then propagated under a mutation rate of *μ* = 10^−^^5^ per base, and selection. Samples were collected from the data spanning sets of three, two, and one locus, each set being sampled at a constant depth N_s_ from the initial population, and then at timepoints corresponding to 1, 3, and 5 days after the beginning of the simulation. These simulations were used to construct a set of full haplotypes, as for the real data, using which an inference of selection was made. Sampling was performed using a standard multinomial model, with an appropriately high value of *C* = 10^6^ being used for inference purposes.

## Supplementary Material

Supplementary text, figures S1–S10, and tables S1 and S2 are available at *Molecular Biology and Evolution *online (http://www.mbe.oxfordjournals.org/).

Supplementary Data
