# Supplementary material for: Fitness Inference from Short-Read Data: Within-Host Evolution of a Reassortant H5N1 Influenza Virus
Source: Mol Biol Evol. 2015 Aug 4;32(11):3012–26. doi: 10.1093/molbev/msv171 (PMC4651230; doi:10.1093/molbev/msv171)
Supplement: Supplementary Data [file supp_32_11_3012__index.html]

Fitness Inference from Short-Read Data: Within-Host Evolution of a Reassortant H5N1 Influenza Virus — Fitness Inference from Short-Read Data: Within-Host Evolution of a Reassortant H5N1 Influenza Virus — Supplementary Data 

# Fitness Inference from Short-Read Data: Within-Host Evolution of a Reassortant H5N1 Influenza Virus

## Supplementary Data

files

- Supplementary Data - txt file
- Supplementary Data - pdf file
- Supplementary Data - txt file
