# Supplementary material for: Fitness Inference from Short-Read Data: Within-Host Evolution of a Reassortant H5N1 Influenza Virus
Source: Mol Biol Evol. 2015 Aug 4;32(11):3012–26. doi: 10.1093/molbev/msv171 (PMC4651230; doi:10.1093/molbev/msv171)
Supplement: Supplementary Data [file supp_msv171_Supplementary_Information.pdf]

Supporting Text

1. Ability to identify hitchhiking given changes in multiple allele frequencies
2. Effect of mis-specification of the mutation rate in inferring haplotype fitnesses I: different real mutation rates, constant rate used for inference
3. Inferences of selection for individual replicate animals
4. Comparison of results from independent inferences of fitness landscapes
5. Location in the protein structure of amino acids associated with non-synonymous changes identified as being under selection
6. Effect of mis-specification of the mutation rate in inferring haplotype fitnesses II: unknown real mutation rate, different rates used for inference
7. Ability to detect and reconstruct low-frequency haplotypes
8. Estimating an effective depth of sampling
9. Code availability
10. Details of inferred partial haplotype frequencies

1. Ability to identify hitchhiking given changes in multiple allele frequencies

Our model showed a good ability to identify hitchhiking from situations in which multiple independent alleles changed in frequency. A system was simulated in which selection at a single allele caused the hitchhiking of multiple alleles. Simulated haplotype frequencies and inferred fitness parameters are shown in Supporting Figure S1. At a standard level of sampling, false inferences of selection were identified in three of ten systems; increasing the depth of sampling removed such false inferences.

2. Effect of mis-specification of the mutation rate in inferring haplotype fitnesses I: different real mutation rates, constant rate used for inference

Simulated data was used to evaluate the effect of using an incorrect mutation rate to infer fitness values from data. Ten samples from a simulated population were used to perform independent inferences of selection from a population evolving on a fitness landscape with mutation rates of 10^{-6}, 10^{-5}, and 10^{-4} per nucleotide, and for a case with a transition rate of $2x10^{-5}$ and a transversion rate of $2x10^{-6}$. In each case inferences of selection were performed with an assumed mutation rate of 10^{-5}. Changes in the mutation rate affected which haplotypes reached higher frequencies in the population; at higher mutation rates positively selected haplotypes that did not initially exist in the population were quicker to emerge. However, the performance of the method was not greatly changed; mean R^2 correlations between the real and inferred haplotypes varied between 0.87 to 0.92, with a mean value of 0.90 where the correct mutation rate was used for inference (Supporting Figure S2).

3. Inferences of selection for individual replicate animals

Within the main text, selection was inferred across sets of animals, under the assumption of an underlying common fitness landscape. Further calculations were

performed on data from individual animals to check for underlying consistency. Inferences are shown in Supporting Figures S3 and S4.

Within different animals, different alleles were inferred as being under selection. Variation in whether or not selection is inferred for a specific allele often reflected clear differences in the observed data from each population, which may arise due to stochastic effects in the establishment of the initial viral population. For example, the A339G mutation was observed in the input population at a frequency of 0.025%, representing 4 out of 16202 observations. At such a frequency, stochasticity in the earliest stages of infection is likely able to determine whether or not the allele establishes in the infecting population and at what effective initial frequency. In animal 991, this allele grew to a frequency of 52.5% in the population at the time five days post infection (16700 out of 31800 observations), while in animal 3501, the equivalent time point showed an observed allele frequency of only 0.07% (12 out of 16894 observations). Consequently, in animal 991, selection was inferred to act upon this allele, whereas no such selection could be inferred from the data for animal 3501.

4. Comparison of results from independent inferences of fitness landscapes

Selection was inferred in both experiments for both the G496T and the G738A mutations. In order to compare the inferred parameters, it must be noted that in the population of experiment 2, the C1020T mutation is fixed, such that epistatic effects involving this allele must be accounted for in any comparison. Thus, the fitness parameter corresponding to G738A in experiment 2 of 0.93 should be compared to the sum of parameters G738A and $\chi_{738,1020}$ from experiment 1, equal to 0.99. Inferred parameters for G496T are more variable, at 0.72 and 1.07 from experiments 1 and 2 respectively, though the combined sum of fitness parameters G496T+G738A from experiment 2, at 1.65, was very close to the equivalent sum of parameters G496T+G738A+$\chi_{738,1020}$+$\chi_{496,738,1020}$ from experiment 1, equal to 1.63. A comparison of inferred haplotype fitnesses f_a from each experiment is provided in Supporting Figure S5.

5. Location in the protein structure of amino acids associated with non-synonymous changes identified as being under selection

Of the nucleotide changes identified as being under selection, a high proportion were non-synonymous in nature. Associated changes in amino acids were generally clustered around the head of the HA molecule; their locations are shown in Supporting Figure S6.

6. Effect of mis-specification of the mutation rate in inferring haplotype fitnesses II: unknown real mutation rate, different rates used for inference

Inferences for the data from experiment 1 were repeated using different mutation rates within the inference model, including mutation rates of 10^{-6}, 10^{-5}, and 10^{-4} per nucleotide, and a case with a transition rate of $2x10^{-5}$ and a transversion rate of

$2x10^{-6}$. Among haplotypes reaching an inferred frequency of 1% in the default population, inferred haplotype fitnesses showed a generally good correlation with the real values (Supporting Figure S7). The topology of the fitness landscape presented in the main text was not greatly changed (Supporting Figure S8).

Under a high mutation rate, haplotypes under strong positive selection are created rapidly via mutation, and rise to significant frequencies in the population. Under an additive model with many positively selected alleles, there are a large number of such haplotypes. Where these haplotypes are not observed in the real population, then under a high mutational rate additional negative epistasis terms are required to prevent their emergence in the model.

It should be noted that the differences in inferred values shown here cannot easily be converted into simple error bars for parameters, as the optimal values obtained under different assumed mutation rates have differing associated likelihoods and BIC scores. A full calculation of error bars would require modification of the code to optimize the mutation rate (or rates, noting that they are likely to be non-homogeneous). This was not possible given the current implementation of the code, and the available computational resources.

7. Ability to detect and reconstruct low-frequency haplotypes

Simulations indicated that our mechanism for haplotype construction was likely to capture haplotypes existing at a frequency of 2% or higher in the population, given the available depth of sequencing. In each simulation, one hundred sets of haplotypes were generated, consisting of random sets of A and G nucleotides, in which the frequency of the haplotype i was equal to 2^{-i}, for i = 1, 2, 3, etc. Random partial haplotypes were drawn from this population according to the alleles spanned by the data collected for ferrets 13 and 991, in numbers equal to the actual depth of sampling. Next, haplotypes were reconstructed using the algorithm described in the main text; the frequency of the largest haplotype in the real population that was not described was then recorded.

Where eight-locus haplotypes were reconstructed, as for ferret 991, the most frequent six haplotypes were recovered in every case, representing reconstruction of all haplotypes at frequencies of 1.6% or higher. Where twelve-locus haplotypes were reconstructed, as for ferret 13, the most frequent six haplotypes were reconstructed in 85% of cases (Supporting Figure S10). We note that, within the analyses performed, haplotypes were reconstructed from data collected across multiple infections; a haplotype would only need to be exist at sufficient frequency in a single population in order to be included in the overall reconstruction.

8. Estimating an effective depth of sampling

Within the calculations performed using the multi-locus data, a Dirichlet multinomial distribution was used to process data, with an inferred over-dispersion parameter of C=64.2199. For the purpose of generating simulated data, random variables were

drawn from a multinomial distribution. For this purpose, we note, very informally, that a multinomial distribution with depth N=65 has an approximately similar shape to the inferred Dirichlet multinomial model.

In a given dimension, the mean and standard deviation of the respective dimensional distributions are given by the equivalent statistics of the binomial and beta-binomial models. To account for different sampling depths, we scale each distribution by N, creating distributions on the space

$$\left\{0, \frac{1}{N}, \ldots, \frac{N-1}{N}, 1\right\}$$

as follows:

$$f_B = P\left(x = \frac{k}{N}\right) = \binom{N}{k} p^k (1-p)^{N-k}$$

$$f_{BB} = P\left(x = \frac{k}{N}\right) = \binom{N}{k} \frac{B(k+\alpha, n-k+\beta)}{B(\alpha, \beta)}$$

Then the mean of f_B is:

$$M_b = \frac{Np}{N} = p$$

Defining parameters:

$$\alpha = Cp$$
$$\beta = C(1-p)$$

the mean of f_{BB} is

$$m_{BB} = \frac{N\alpha}{N(\alpha+\beta)} = p$$

Considering standard deviations, we have

$$S_b = \frac{\sqrt{Np(1-p)}}{N} = \frac{\sqrt{p(1-p)}}{\sqrt{N}}$$

while

$$S_{bb} = \frac{\sqrt{\frac{C^2 N(C+N)p(1-p)}{C^2(1+C)}}}{N}$$

We then note that as N tends to infinity

$$S_{bb} \to \frac{\sqrt{\frac{N^2 p(1-p)}{(1+C)}}}{N} = \sqrt{\frac{p(1-p)}{1+C}} = \frac{\sqrt{p(1-p)}}{\sqrt{1+C}}$$

such that equality between this statistic of the distributions is achieved when N=1+C. In the extension to the multinomial case, the above calculation applies to each dimension of the multidimensional distribution. A multinomial distribution with sampling depth N=65 was used for simulations described in the main text.

9. Code availability

Code generated for this project is available from the web address http://websvc.gen.cam.ac.uk/~cjri2/SupportingCode.tar.gz . Code was written in C++ and Mathematica and tested with Mac OS X 10.9.

10. Details of inferred partial haplotype frequencies

Full details of inferences are given in the Supporting Figures S11 and S12. These figures are provided in Mathematica .cdf format, and may be viewed using the freely available Mathematica CDF Player software.

Supporting Figure S11: Inferred haplotype frequencies for experiment 1. Inferred frequencies are shown as lines coloured by partial haplotype. Vertical bars are centered on the observed partial haplotype frequencies; the length of the bar shows the standard deviation of each frequency given the inferred level of uncertainty in the data. Sliding bars control for which animal data are shown, and which set of partial haplotypes are displayed.

Supporting Figure S12: Inferred haplotype frequencies for experiment 2. Inferred frequencies are shown as lines coloured by partial haplotype. Vertical bars are centered on the observed partial haplotype frequencies; the length of the bar shows the standard deviation of each frequency given the inferred level of uncertainty in the data. Sliding bars control for which animal data are shown, and which set of partial haplotypes are displayed

.

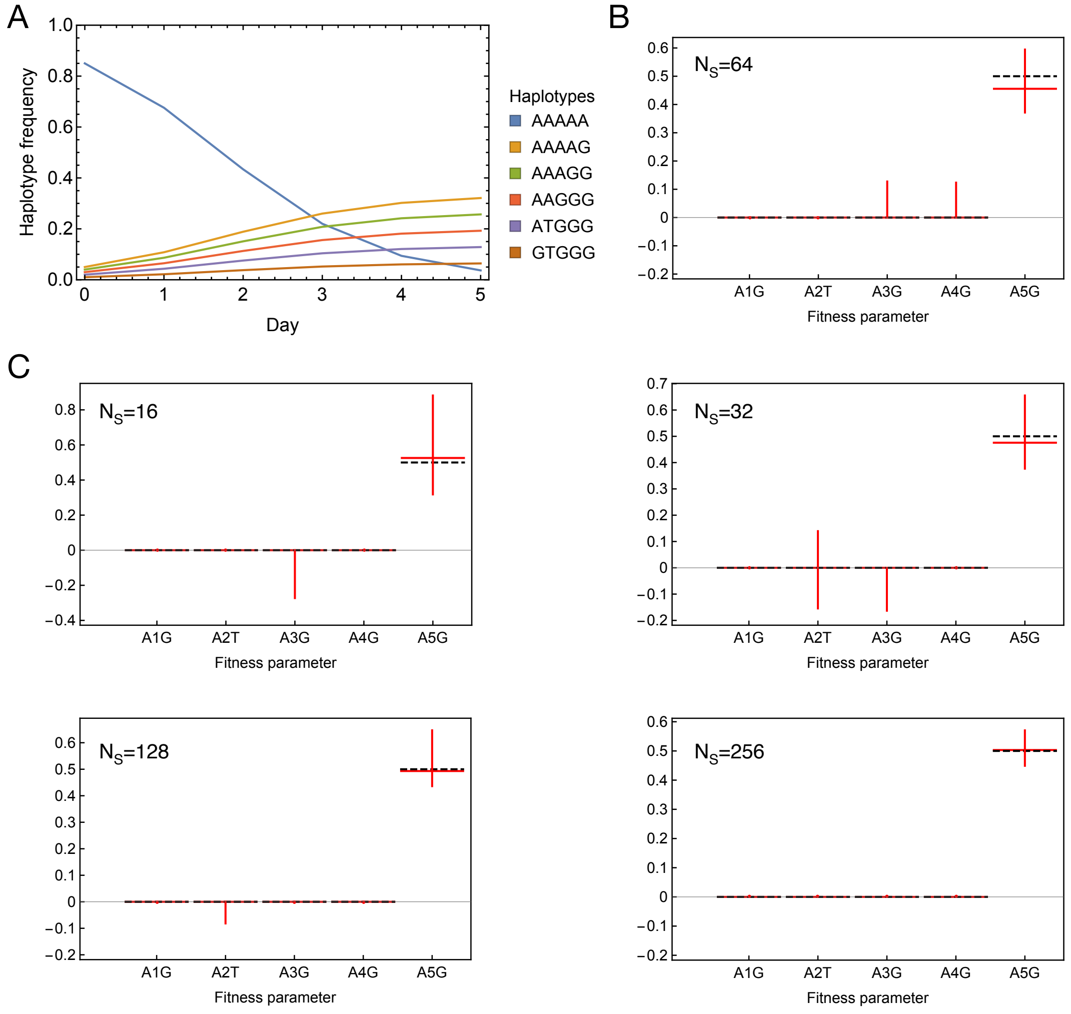

Supporting Figure S1: Inference of selection in a system with hitchhiking. A. Selection for the allele G at the fifth position of each haplotype causes the movement of all allele frequencies via hitchhiking. **B.** Inferred fitness parameters with a sample depth of 64. Horizontal black dashed lines show the correct selection parameters. Median inferences of selection across 10 replica samples are shown as horizontal solid red lines; vertical red lines show the range of inferred parameters. **C.** Inferred fitness parameters given different sampling depths. Higher sampling depths reduce the variance from the correct inference.

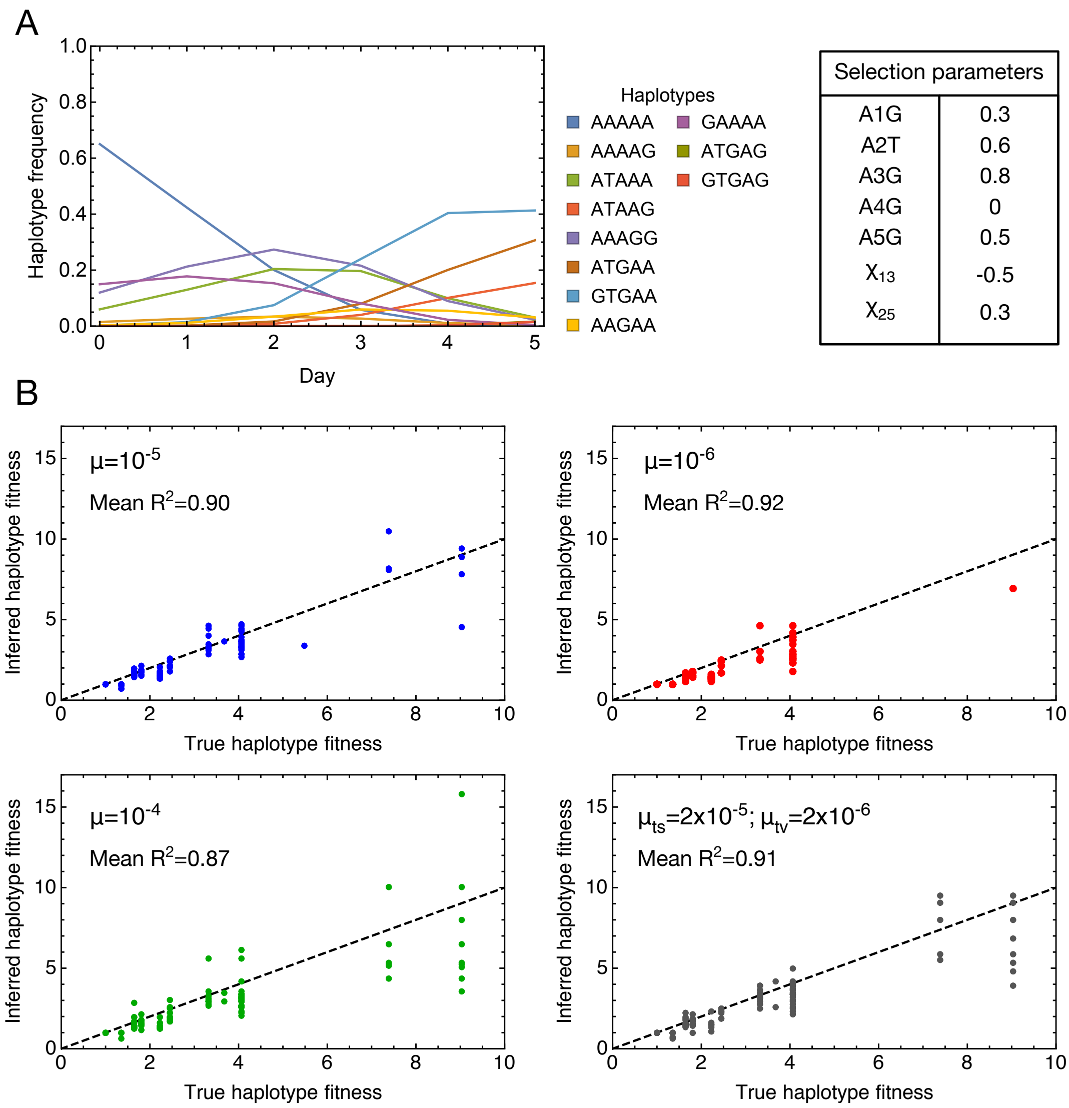

Supporting Figure S2: Inferences of haplotype fitnesses for simulated systems with different mutation rates: A. Changes in haplotype frequencies and selection parameters for a simulated system, here shown with a simulated mutation rate of 10^{-5} per nucleotide per generation. **B.** Inferred haplotype fitness values for haplotypes inferred to reach a frequency of at least 1% at some time during the evolution of the system. Results are shown for different simulated mutation rates μ. A mutation rate of 10^{-5} was used in the inference model in every case.

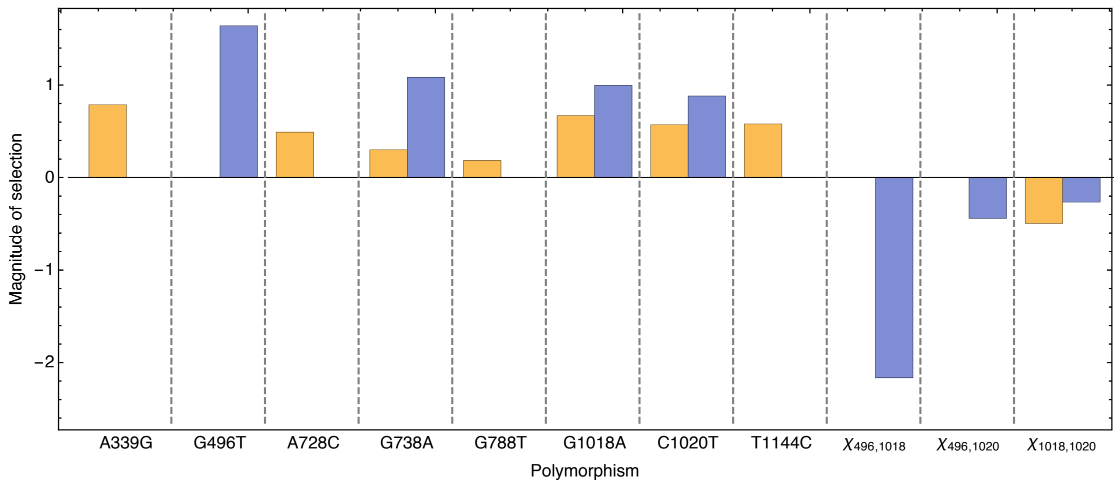

Supporting Figure S3: Inferred parameters for replicate ferrets from the first experiment. Inferred parameters are shown for ferrets F991 (yellow) and F3501 (blue). Where selection was inferred for the same allele in both animals, the magnitudes of selection are similar.

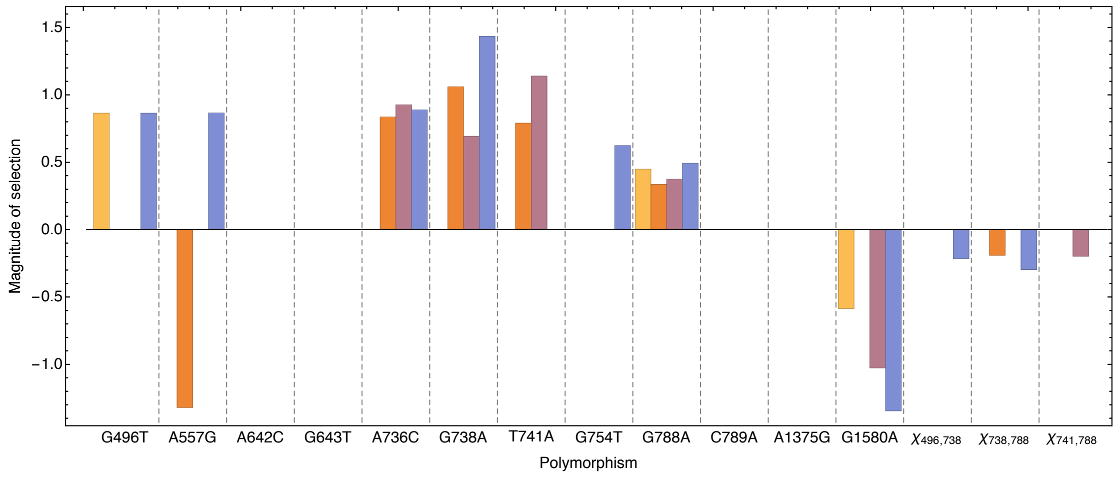

Supporting Figure S4: Inferred parameters for replicate ferrets from the second experiment. Inferred parameters are shown for ferrets F13 (yellow), F15 (orange), F17 (purple), and F21 (blue). Where selection was inferred for the same allele in both animals, the magnitudes of selection are largely similar. Deviations in magnitude are associated with the inference, or non-inference, of epistasis involving a specific allele.

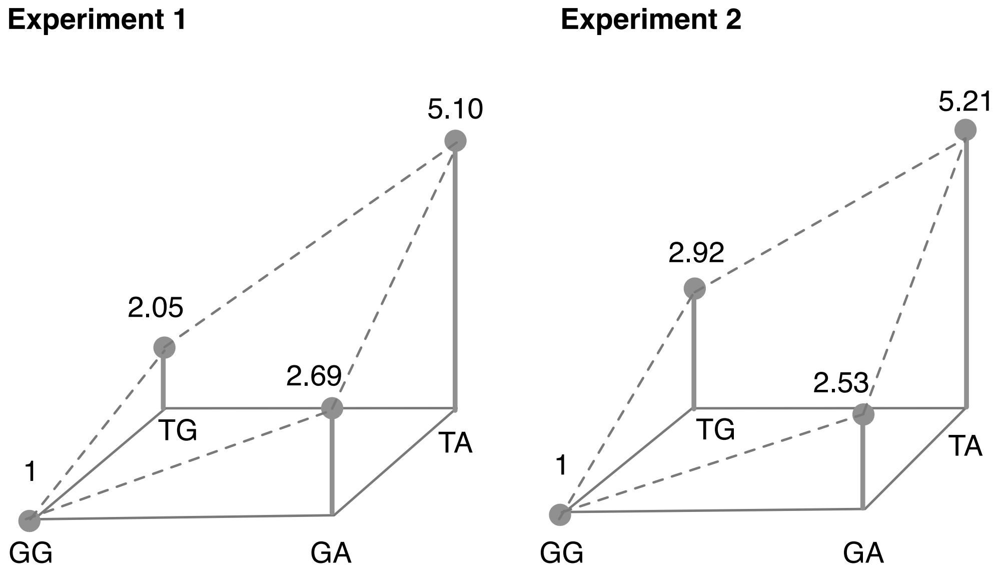

Supporting Figure S5: Comparison of haplotype fitnesses f_a at loci identified as having alleles under selection in both experiments. The independently derived fitness landscapes show inferences made for each of the potential two-locus haplotypes at loci 496 and 738 relative to the consensus GG haplotype.

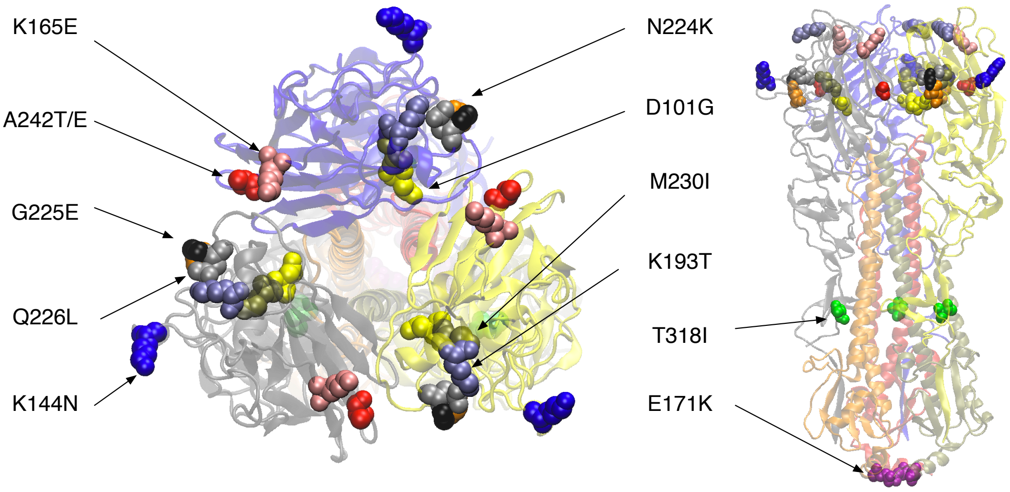

Supporting Figure S6: Location of non-synonymous mutations inferred as being under selection. Amino acids are shown in each of the three HA proteins in the trimer structure, shown from the top (left) and side (right). The majority of substitutions occur in the head region of the protein. Structural information is taken from the PDB structure 2FK0.

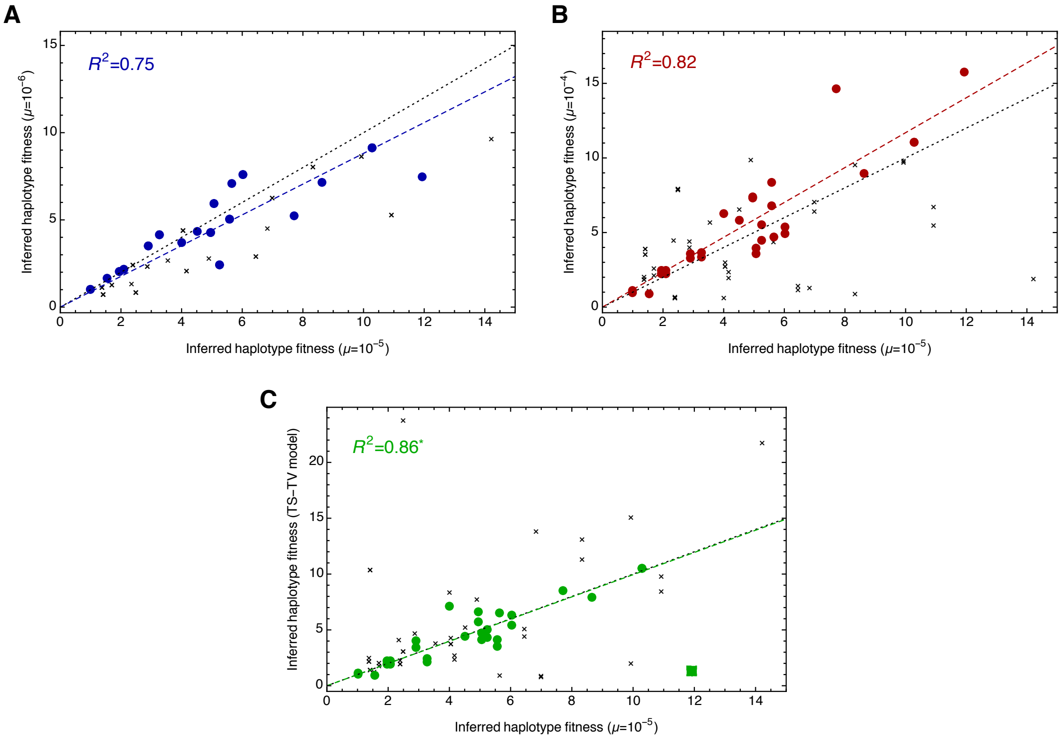

Supporting Figure S7: Variance in inferred fitness values given changes in the mutation rate used for inference. Inferred haplotype fitnesses from the first experiment given the default mutation rate of μ=10^{-5} compared to those inferred with (A) μ=10^{-6} (blue), (B) μ=10^{-4} (red), and (C) with a significant transition-transversion (μ_{ts}=2x10^{-5}; μ_{tv}=2x10^{-6}) (green). Fitnesses for haplotypes that were inferred to reach a frequency of 1% or greater are shown in colour; fitnesses for other haplotypes are shown as black crosses. Coloured dotted lines show respective fits to the data via linear regression; the black dotted line is that of perfect agreement with the values inferred using μ=10^{-5}. Regression coefficients are calculated for high frequency haplotypes; in the transition-transversion case a single outlier, marked with a square, was excluded from the calculation.

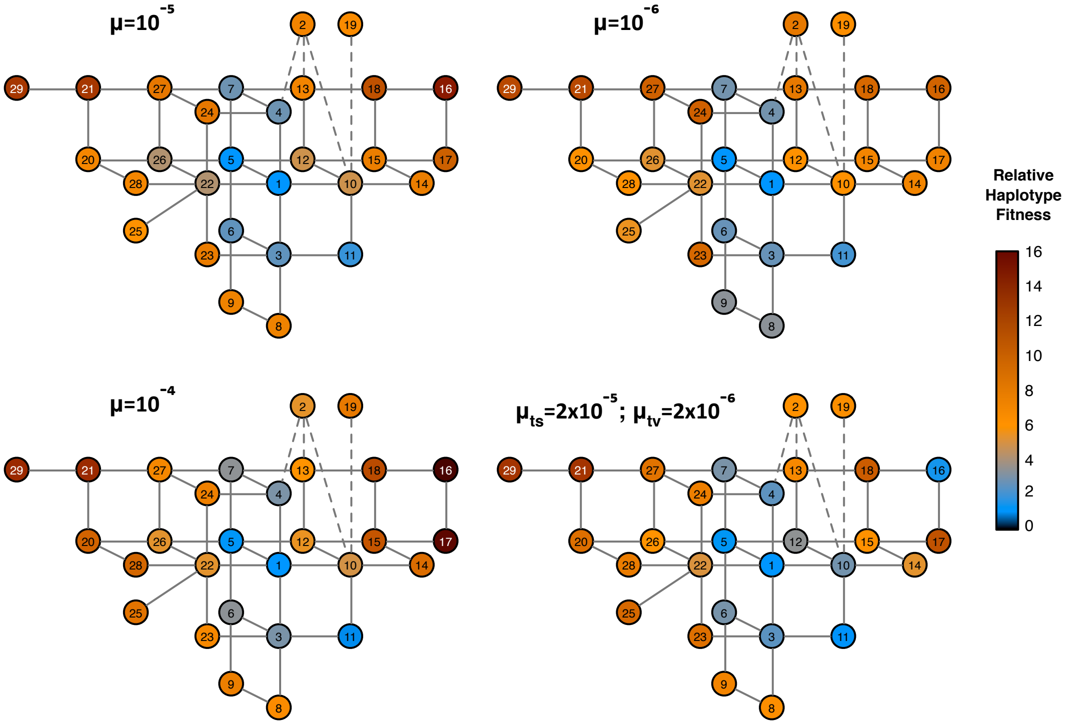

**1**	A G A G G G C T	**9**	A G A A T G T T	**16**	G G A A T A C C	**23**	A T A G G G T T
**2**	G G C G G A C T	**10**	G G A G G G C T	**17**	G G A A T G C C	**24**	A T A G G A C T
**3**	A G A G G G T T	**11**	G G A G G G T T	**18**	G G A A T A C T	**25**	A T A G G G C C
**4**	A G A G G A C T	**12**	G G A G T G C T	**19**	G G C G G G C C	**26**	A T A G T G C T
**5**	A G A G T G C T	**13**	G G A G T A C T	**20**	A T A A T G C T	**27**	A T A G T A C T
**6**	A G A G T G T T	**14**	G G A A G G C T	**21**	A T A A T A C T	**28**	A T A A G G C T
**7**	A G A G T A C T	**15**	G G A A T G C T	**22**	A T A G G G C T	**29**	A T C A T A C T
**8**	A G A A G G T T						

Supporting Figure S8: Inferred fitness landscapes at different mutation rates. Across a 100-fold change in the model mutation rates, few qualitative changes to the inferred fitness landscape were observed. Fitness values are shown for haplotypes inferred to reach a frequency of 1% in at least one animal, according to the inference made at the default mutation rate.

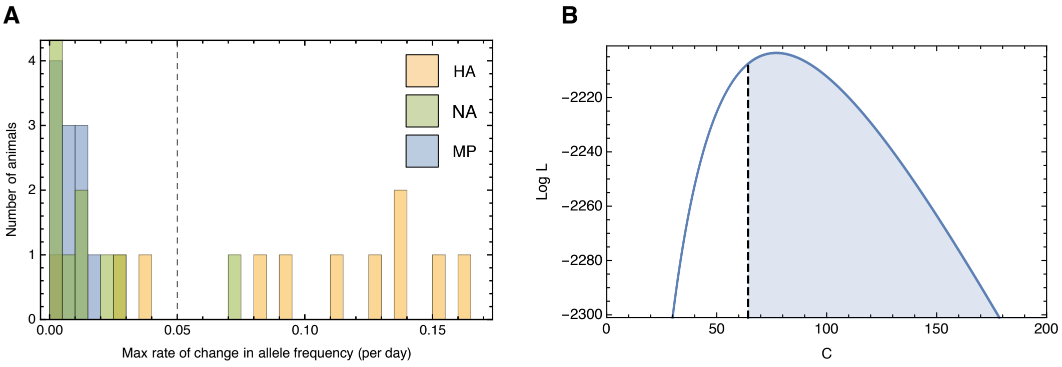

Supporting Figure S9: Inference of noise in the data. A. Identification of systems evolving in a potentially neutral manner. A conservative cutoff of a maximum allele frequency change of 5% per day divided the majority of HA populations from the majority of NA and MP populations. **B.** Likelihood of the observed partial haplotypes from the potentially neutral systems under a model of neutral evolution plus noise. The parameter C characterizes the extent of noise; lower values indicate increased noise. The vertical line at C=64.2199 denotes a conservative estimate of this parameter.

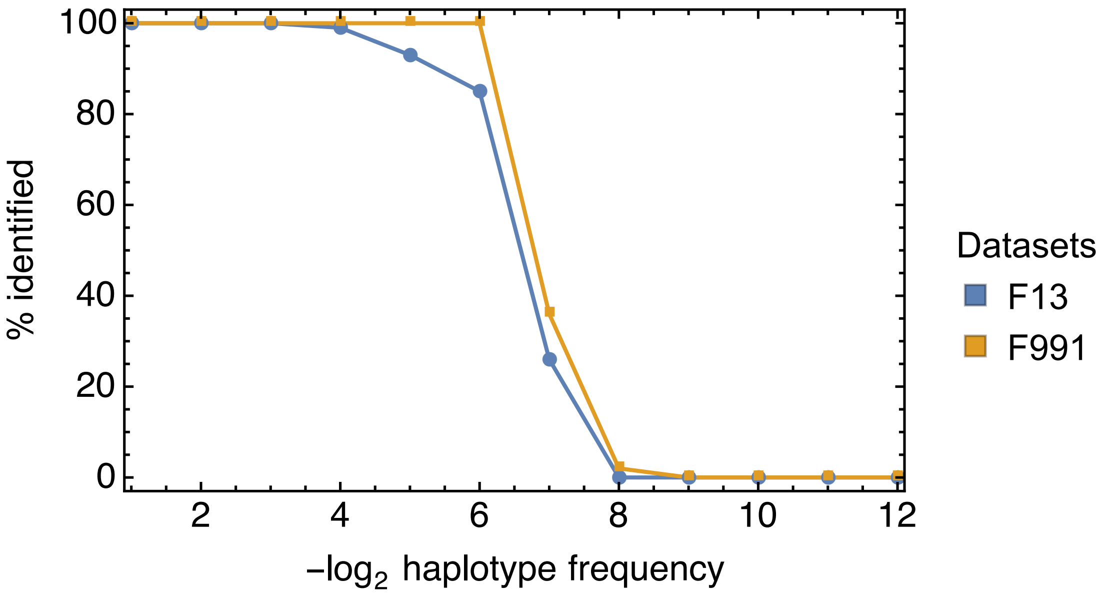

Supporting Figure S10: Performance of the haplotype reconstruction algorithm: Percentage of simulated occasions upon which haplotypes of given underlying frequencies were reconstructed, given simulated data. Here a frequency of 2^-6 indicates that all haplotypes at frequencies 2^{-6} and above were reconstructed given simulated short-read data matching that for a specific animal. Percentages were calculated over 100 simulations.
